# Interstitial lung disease associated with chronic liver disease

**DOI:** 10.5578/tt.20239612

**Published:** 2023-12-07

**Authors:** Övgü Velioğlu Yakut, Miraç ÖZ, Öznur YILDIZ, Özlem Özdemir Kumbasar

**Affiliations:** 1 Department of Chest Diseases, Ankara University Faculty of Medicine, Ankara, Türkiye

## Abstract

**ABSTRACT**

**
Interstitial lung disease associated with chronic liver disease
**

*
It is important to make the differential diagnosis of restrictive changes associ- ated with hepatic hydrothorax or hepatopulmonary syndrome seen in the later stages of chronic liver diseases and restrictive changes associated with interstitial lung disease. Lymphocytic interstitial pneumonia (LIP) is in the rare idiopathic interstitial pneumonia subgroup of interstitial lung diseases. LIP is a rare disease, and its incidence is unknown. LIP is characterized by infiltra- tion of the alveolar interstitium with lymphocytes, plasma cells, and histio- cytes. The etiology of LIP includes idiopathic causes, rheumatological diseas- es, immune deficiencies, viral infections, and drug-related causes. Chronic liver diseases are also rarely included in the etiology of LIP. A 75-year-old male patient who was followed up for liver cirrhosis presented with dyspnea. He had hypoxemia in the arterial blood gas. In the thorax and abdominal computed tomography, irregular reticulations in bilateral lungs, ground-glass opacities, and scattered air cysts in both lung parenchyma, chronic liver parenchymal disease, splenomegaly, chronic portal vein thrombosis were determined. Clinical and radiological changes in the patient were evaluated in favor of interstitial lung disease. Although histopathological diagnosis could not be made, the patient whose radiological pattern was compatible with LIP was evaluated together with clinical findings and was accepted as lympho- cytic interstitial pneumonia. He was evaluated in terms of diseases that could cause LIP. He was accepted as LIP due to chronic liver disease. Although histopathological examination is the gold standard for the diagnosis, a biopsy could not be performed in our case. Radiological and clinical findings were considered sufficient for the diagnosis of LIP. Chronic viral hepatitis and cir- rhosis are also present in the etiology of LIP. Our case is presented as an example in the literature because it is a case of LIP due to chronic liver dis- ease, and it is rare.
*

**Key words:** *Interstitial lung disease; chronic hepatitis; chronic liver disease; lymphocytic interstitial pneumonia; cirrhosis
*

**ÖZ**

**
Kronik karaciğer hastalığına eşlik eden interstisyel akciğer hastalığı
**

*
Kronik karaciğer hastalıklarının ilerleyen dönemlerinde görülen hepatik hidrotoraks veya hepatopulmoner sendrom ilişkili görülen restriktif değişiklikler ile interstisyel akciğer hastalığına bağlı görülen restriktif değişikliklerin ayırıcı tanısı yapılması önem taşımaktadır. Lenfositik interstisyel pnömoni (LİP), interstisyel akciğer hastalıklarının nadir görülen idiyopatik interstisyel pnömoni alt grubunda yer alır. LİP nadir görülen bir hastalıktır ve insidansı net olarak bilinmemektedir. LİP, alveolar interstisyumun lenfositler, plazma hücreleri ve histiyositlerle infiltrasyonu ile karakterizedir. LİP etiyolojisinde idiyopatik nedenler, romatolojik hastalıklar, immün yetmezlikler, viral enfeksiyonlar ve ilaca bağlı nedenler yer alır. Kronik karaciğer hastalıkları LİP etiyolojisinde nadiren yer alır. Karaciğer sirozu nedeniy- le takip edilen 75 yaşında erkek hasta tarafımıza nefes darlığı şikayetiyle başvurdu. Arter kan gazında hipoksemi görüldü. Toraks ve batın bilgisayarlı tomografisinde bilateral akciğerlerde düzensiz retikülasyonlar, buzlu cam opasiteleri ve her iki akciğer parankiminde dağınık hava kistleri, kronik karaciğer parankim hastalığı, splenomegali, kronik portal ven trombozu saptandı. Hastadaki klinik ve radyolojik değişiklikler interstisyel akciğer hastalığı lehine değerlendirildi. Radyolojik paterni LİP ile uyumlu olan hasta histopatolojik tanı konulamamasına rağmen klinik bulguları ile birlikte değerlendirilerek lenfositik interstisyel pnömoni olarak kabul edildi. LİP’e neden olabilecek diğer hastalıklar açısından da değerlendirilen hastada etiyolojisi kronik karaciğer hastalığı olarak düşünüldü. LİP tanısında histopatolojik inceleme altın standart olmasına rağmen bizim olgumuzda biyopsi yapılamadı. LİP tanısı için radyolojik ve klinik bulgular yeterli kabul edildi. Lenfositik interstisyel akciğer hastalığının etiyolojisinde kronik viral hepatitler ve siroz da yer almak- tadır. Olgumuz kronik karaciğer hastalığına bağlı bir LİP olgusu olması ve nadir görülmesi nedeniyle literatüre örnek olarak sunulmuş- tur.
*

**Anahtar kelimeler:** *İnterstisyel akciğer hastalığı; kronik hepatit; kronik karaciğer hastalığı; lenfositik interstisyel pnömoni; siroz
*

## INTRODUCTION

The coexistence of lung and liver diseases can be evaluated under three main headings. In the first group, we can count diseases affecting both the lungs and liver, such as alpha 1 antitrypsin deficiency, cys- tic fibrosis and sarcoidosis. Lung pathologies that can be found together with chronic liver diseases make up the second group. Diseases such as obstructive lung diseases (asthma, COPD), interstitial lung dis- eases, accompanying pulmonary nodules can be counted as examples of this group. In the third group, pulmonary complications secondary to end-stage liver disease and portal hypertension can be listed. Hepatopulmonary syndrome, portopulmonary hyper- tension, hepatic hydrothorax can be given as exam- ples of this group. It is important to make the differ- ential diagnosis of restrictive changes associated with hepatic hydrothorax or hepatopulmonary syndrome seen in the later stages of chronic liver diseases and restrictive changes associated with interstitial lung disease (1). Lymphocytic interstitial pneumonia (LIP) is in the rare idiopathic interstitial pneumonia sub- group of interstitial lung diseases. LIP is characterized by infiltration of the alveolar interstitium with lym- phocytes, plasma cells, and histiocytes (2). Idiopathic causes, rheumatological diseases (Sjögren’s syn- drome, rheumatoid arthritis, systemic lupus erythe- matosus, celiac disease, Myasthenia gravis, perni- cious anemia, chronic active hepatitis, biliary cirrho- sis), immunodeficiencies (common variable immuno-

deficiency), viral infections (HIV, EBV, HHV8, HTLV- 1), dysproteinemias, bone marrow transplantation, Castleman disease, use of diphenylhydantoin, Waldenstrom hypergammaglobulinemia, pulmonary amyloidosis, surfactant protein C deficiency, and drug-related causes take part in the etiology of LIP (3-5). In this report, it was aimed to present a case that was considered to have developed lymphocytic interstitial pneumonia due to chronic viral hepatitis. Chronic liver diseases should be remembered to explain LIP etiology. Our case presentation is import- ant for literature because of its rare occurrence.

## CASE REPORT

A 75-year-old male patient was admitted to our clinic with dyspnea. On physical examination, auscultation was bilaterally normal. Oxygen saturation in room air was 88%. He had smoked a pack of cigarettes daily for 55 years and had heavy alcohol consumption. He was diagnosed with active viral hepatitis-B 28 years ago and had interferon-alpha therapy for eight months. He was diagnosed with liver cirrhosis 20 years ago and followed up due to cirrhosis. Tenofovir treatment was initiated. Partial oxygen pressure

(PaO2) was 40 mmHg in the arterial blood gas. Nasal oxygen therapy was started at 2 lt/min. Pulmonary

function tests were performed and forced vital capacity (FVC), FEV1/FVC were measured and determined respectively FEV1: 65% (1.75 lt), FVC: 79% (2.85 lt), FEV1/FVC: 61%. Carbon monoxide diffusion capacity (DLCO) was found 47%, decreased.

In laboratory findings, total bilirubin was 2.39 mg/ dL, indirect bilirubin was 1.84 mg/dL, and other liver function tests, erythrocyte sedimentation rate, and C-reactive protein were normal. Viral serological markers were evaluated such as anti-HDV, HBsAg, anti-HBs, HBV DNA. Only anti-HDV was determined as positive. Rheumatoid factor (RF) level was 163 IU/ mL, and anti-CCP was found negative. Anti-nuclear antibody (ANA) showed weak positive results, and nRNP/Sm was determined positive. Other immunological markers were found negative. He was consulted for rheumatological diseases. T, salivary gland biopsy was performed for the presence of sialadenitis, and nonspecific inflammation signs were observed in the pathological examination.

Posteroanterior chest radiography was compatible with diffuse reticular densities that became apparent towards the lower zones (Figure 1). As a result of

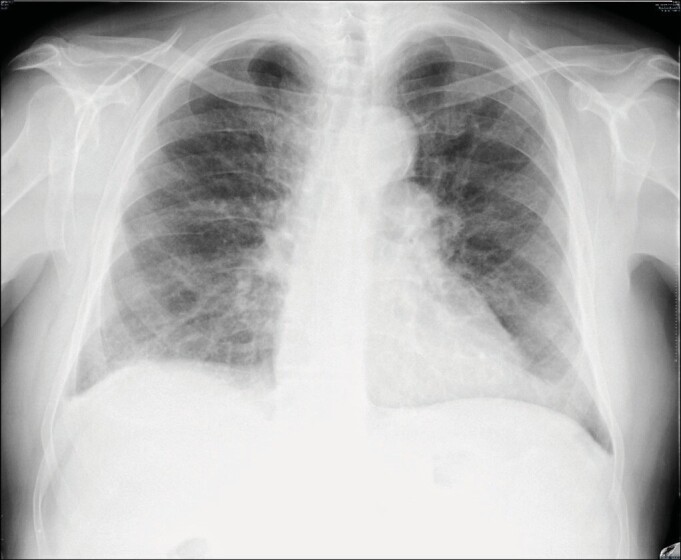

**Figure 1.** Posteroanterior chest radiography shows flattening of the right diaphragm, prominence in the pulmonary conus, enlargement in the right paratracheal area, diffuse reticular densities prominent towards the lower zones.

chest computed tomography (CT) findings, it was accepted as LIP because of irregular reticulations, ground-glass areas, and scattered air cysts (Figure 2). Chronic liver parenchymal disease, splenomegaly, and chronic portal vein thrombosis were found on the liver areas included in the chest CT scan (Figure 3). The patient had esophageal varices due to liver cirrhosis. He had a history of band ligation and sclerotherapy via esophageal varicose bleeding. Chronic portal vein thrombosis and esophageal varices were considered to be related to cirrhosis complications.

## DISCUSSION

Chronic liver diseases such as chronic active hepatitis and primary biliary cirrhosis can affect the lung parenchyma through abnormal connections between the portal and pulmonary veins (4). Fibrosing alveolitis, bronchiolitis obliterans, and pleurisy are lung pathologies that can be seen in autoimmune hepatitis (4-6).

Although 5% of LIP cases are asymptomatic, chronic cough and progressive dyspnea are the most common complaints. Respiratory symptoms are present in most patients at diagnosis. Weight loss, fever, night sweats, pleuritic chest pain, muscle weakness, arthralgia are other systemic symptoms. On physical examination, rales, clubbing (10%), hepatosplenomegaly, lymphadenopathy, parotitis, and arthritis can be seen due to the etiology. Clubbing is a common physical finding, peripheral and mediastinal lymphadenopathy, and splenomegaly are scarce (3). In our case, increased dyspnea in the last six months was the most important symptom, auscultation findings of the respiratory system was normal and other systems findings were also considered normal.

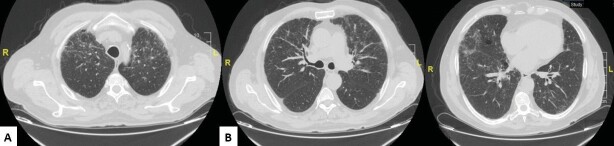

**Figure 2.** In chest computed tomography, irregular reticulations and ground-glass areas in both lungs **(A-C)**, scattered air cysts in both lung parenchyma **(C)**.

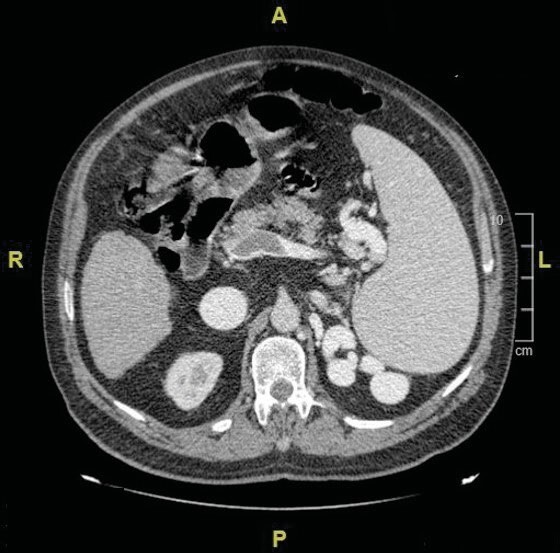

**Figure 3.** Chronic liver parenchymal disease, splenomegaly, chronic portal vein thrombosis.

Normal or decreased partial oxygen pressure can be measured in arterial blood gas analysis. Restrictive ventilatory pattern is often seen in pulmonary function tests, also decreased or normal lung volumes and decreased DLCO are compatible with this pattern. In our case, there was moderate hypoxemia in the arterial blood gas and decreased DLCO.

Radiologically, ground glass, reticular or reticulonodular opacities, subpleural nodules, peribronchovascular cystic lesions, centrilobular nodules, interlobular septal thickening can be seen on chest CT. Bronchiectasis and honeycomb appearance are rare. Perivascular cystic lesions can be a single sign. Cysts present usually in the lower lobes and peribronchovascular areas (6-8). It is stated that the most common findings in chest CT images for LIP diagnosis are ground glass appearance, centrilobular nodules, subpleural small nodules, bilateral reticular and reticulonodular opacities in the lower zones. Thickening of the bronchovascular branches and interlobular septa, presence of air cysts and lymphadenopathy are other imaging findings (9). In our case, chest CT findings were accepted as LIP because of irregular reticulations, ground-glass areas, and scattered air cysts. The diagnosis of our patient was made clinically and radiologically, and other

causes in the etiology, including immunological pathologies, were ruled out.

Bronchoalveolar lavage (BAL) findings are nonspecific, and lymphocyte dominance is approximately 30%. Alveolar septal infiltration of lymphocytes, plasma cells, and histiocytes on pathological examination is important for LIP diagnosis (10). Bronchoscopy and bronchoalveolar lavage could not be taken in our case due to the apparent hypoxemia.

Diffuse air cysts were accepted to be compatible with LIP radiologically. Histopathological examination is required for a definitive diagnosis. Biopsy could not be performed for the definitive diagnosis, and the patient was accepted as LIP with clinical and radiological findings. As in the study of Koulaouzidis et al., the diagnosis was made with appropriate clinical and radiological findings in our case (6).

## CONCLUSION

The diagnosis of LIP consists of clinical, radiological, and pathological findings. LIP diagnosis can be made with sufficient clinical and radiological findings when biopsy cannot be performed. LIP should also be considered in the differential diagnosis of bilateral, diffuse, ground-glass nodular opacity and consolidation with air bronchograms on chest X-ray and CT. Although the diagnosis of LIP in our case could not be evaluated histologically, the radiological and clinical findings were found to be compatible with LIP, so it was accepted as lymphocytic interstitial pneumonia due to chronic liver disease, and the patient was followed up in our clinic for lung involvement.

## CONFLICT of INTEREST

The authors have no conflict of interest to declare.

## AUTHORSHIP CONTRIBUTIONS

Concept/Design: ÖY

Analysis/Interpretation: ÖVY, MÖ, ÖY, ÖÖK Data acqusition: ÖVY, MÖ, ÖY, ÖÖK Writing: ÖVY, MÖ

Clinical Revision: MÖ, ÖY, ÖÖK Final Approval: ÖVY, MÖ, ÖY, ÖÖK

## REFERENCES

1. *
  Raevens S, Boret M, De Pauw M, Fallon MB, Van Vlierberghe H. Pulmonary abnormalities in liver disease: Relevance to transplantation and outcome. Hepatology 2021; 74(3): 1674-86. https://doi.org/10.1002/hep.31770*
2. *
  Oliveira DS, Araújo Filho JdA, Paiva AFL, Ikari ES, Chate RC, Nomura CH. Idiopathic interstitial pneumonias: Review of the latest American Thoracic Society/European Respiratory Society classification. Radiol Bras 2018; 51(5): 321-7. https://doi.org/10.1590/0100-3984.2016.0134*
3. *
  Swigris JJ, Berry GJ, Raffin TA, Kuschner WG. Lymphoid interstitial pneumonia: A narrative review. Chest 2002; 122(6): 2150-64. https://doi.org/10.1378/ chest.122.6.2150*
4. *
  Koss MN, Hochholzer L, Langloss JM, Wehunt WD, Lazarus AA. Lymphoid interstitial pneumonia: Clinicopathological and immunopathological findings in
  *

*
18 cases. Pathology 1987; 19(2): 178-85. https://doi. org/10.3109/00313028709077131*

5. *
  Stanley N, Woodgate D. Mottled chest radiograph and gas transfer defect in chronic liver disease. Thorax 1972; 27(3): 315-23. https://doi.org/10.1136/thx.27.3.315*
6. *
  Koulaouzidis A, Karagiannidis A, Prados S, Pattenshetty D, Deramon A, Tan W. Lymphocytic interstitial pneumonitis (LIP)-The liver and the lung. Ann Hepatol 2006; 5(3): 170-
  *

*
1. https://doi.org/10.1016/S1665-2681(19)32003-4*

7. *
  Johkoh T, Müller NL, Pickford HA, Hartman TE, Ichikado K, Akira M, et al. Lymphocytic interstitial pneumonia: Thin-section CT findings in 22 patients. Radiology 1999; 212(2): 567-72. https://doi.org/10.1148/radiolo- gy.212.2.r99au05567*
8. *
  Johkoh T, Ichikado K, Akira M, Honda O, Tomiyama N, Mihara N, et al. Lymphocytic interstitial pneumonia: Follow-up CT findings in 14 patients. J Thorac Imaging 2000; 15(3): 162-7. https://doi.org/10.1097/00005382-*

*200007000-00002*

9. *
  Kurosu K, Yumoto N, Furukawa M, Kuriyama T, Mikata A. Third complementarity-determining-region sequence analysis of lymphocytic interstitial pneumonia: Most cases demonstrate a minor monoclonal population hidden among normal lymphocyte clones. Am J Respir Crit Care Med 1997; 155(4): 1453-60. https://doi.org/10.1164/ ajrccm.155.4.9105093*
10. *
  Panchabhai TS, Farver C, Highland KB. Lymphocytic inter- stitial pneumonia. Clin Chest Med 2016; 37(3): 463-74. https://doi.org/10.1016/j.ccm.2016.04.009*

